# Documentation of best interest by intensivists: a retrospective study in an Ontario critical care unit

**DOI:** 10.1186/1472-6939-11-1

**Published:** 2010-02-10

**Authors:** Mohana Ratnapalan, Andrew B Cooper, Damon C Scales, Ruxandra Pinto

**Affiliations:** 1 University of Toronto, Faculty of Medicine, Department of Health Policy, Management and Evaluation, Department of Management, Toronto, Canada; 2 Department of Critical Care Medicine, William Osler Health System, Brampton Civic Hospital, 2100 Bovaird Drive East, Brampton, Ontario L6R 3J7, Canada; 3 University of Toronto, Faculty of Medicine, Interdisciplinary Division of Critical Care Medicine, Toronto, Canada; 4 Department of Critical Care Medicine, Sunnybrook Health Sciences Centre, 2075 Bayview Avenue, Toronto, Ontario M4N 3M5, Canada

## Abstract

**Background:**

Intensive care physicians often must rely on substitute decision makers to address all dimensions of the construct of "best interest" for incapable, critically ill patients. This task involves identifying prior wishes and to facilitate the substitute decision maker's understanding of the incapable patient's condition and their likely response to treatment. We sought to determine how well such discussions are documented in a typical intensive care unit.

**Methods:**

Using a quality of communication instrument developed from a literature search and expert opinion, 2 investigators transcribed and analyzed 260 handwritten communications for 105 critically ill patients who died in the intensive care unit between January and June 2006. Cohen's kappa was calculated before analysis and then disagreements were resolved by consensus. We report results on a per-patient basis to represent documented communication as a process leading up to the time of death in the ICU. We report frequencies and percentages for discrete data, median (m) and interquartile range (IQR) for continuous data.

**Results:**

Our cohort was elderly (m 72, IQR 58-81 years) and had high APACHE II scores predictive of a high probability of death (m 28, IQR 23-36). Length of stay in the intensive care unit prior to death was short (m 2, IQR 1-5 days), and withdrawal of life support preceded death for more than half (n 57, 54%). Brain death criteria were present for 18 patients (17%). Although intensivists' communications were timely (median 17 h from admission to critical care), the person consenting on behalf of the incapable patient was explicitly documented for only 10% of patients. Life support strategies at the time of communication were noted in 45% of charts, and options for their future use were presented in 88%. Considerations relevant to determining the patient's best interest in relation to the treatment plan were not well documented. While explicit survival estimates were noted in 50% of charts, physicians infrequently documented their own predictions of the patient's functional status (20%), anticipated need for chronic care (0%), or post ICU quality of life (3%). Similarly, documentation of the patient's own perspectives on these ranged from 2-18%.

**Conclusions:**

Intensivists' documentation of their communication with substitute decision makers frequently outlined the proposed plan of treatment, but often lacked evidence of discussion relevant to whether the treatment plan was expected to improve the patient's condition. Legislative standards for determination of best interest, such as the Health Care Consent Act in Ontario, Canada, may provide guidance for intensivists to optimally document the rationales for proposed treatment plans.

## Background

The appropriate use of scarce critical care resources is an important target for quality improvement in our health care system. For example, the final report of the Ontario Critical Care Strategy states "considerable anecdotal evidence exists that Critical Care in Ontario is often provided to patients who do not, or can no longer benefit from this level of care"[1]. In a grounded theory investigation, senior intensive care staff at 16 Ontario intensive care units defined medically futile care as "the use of considerable resources without a reasonable hope that the patient might recover to a state of relative independence, or to be interactive with the environment"[2]. Providers identified family demands and a lack of timely or skilled communication as factors leading to the provision of futile care. Similarly, in a purposive sample of 12 written decisions of the Ontario Consent and Capacity Board (CCB) in which there were conflicts between physicians and substitute decision makers regarding the best interests of incapable patients regarding proposed treatment plans, substitute decision makers frequently appealed to the relevance of God or religion, and emphasized their own values over those of the incapable patient. In contrast, clinicians focused on clinical evidence and their predictions of whether the treatment plan was likely to benefit the patient or to cause harm. The important distinction between a patient's values and previously expressed wishes was emphasized in the summaries of these decisions. The CCB also indicated that substitute decision makers must follow statutory obligations when considering whether the proposed treatment plan would be likely to have a particular effect [3]. In CASCADE, a prospective cohort study of 323 nursing home residents, substitute decision-makers were less likely (adjusted odds ratio 0.33 95% CI 0.17-0.63) to choose burdensome interventions for patients with advanced dementia during the last three months of life when they understood the expected clinical complications of the disease than when they did not[4].

Such findings underscore how important it is for physicians to structure communication with substitute decision makers to address all dimensions of the construct of "best interest" for incapable, critically ill patients. The task is to correctly identify prior wishes and to facilitate the substitute decision maker's understanding of the incapable patient's condition and their likely response to treatment. In Ontario, Canada, Section 21(2) of the *Health Care Consent Act*[5] can be seen as a useful template for structured communication because it encompasses the patient's medical status, the treatment plan, and the substitute decision makers and physicians' constructs of the patient's best interest (Table 1). Section 21(2) is also relevant because it contains the information which must be considered during a CCB review, which may be necessary to resolve a conflict over the appropriateness of a proposed plan of treatment [6]. In this Clinical Ethics Centre initiative, we sought to determine areas needing improvement in our processes of communication by assessing the quality of intensivists' documentation of the goals of care as discussed in their meetings with substitute decision-makers.

**Table 1:** Thematic Classification of Section 21(2) of Ontario, Canada's Health Care Consent Act 1996 §

Medical Status	Treatment Plan at Issue	Substitute Decision Makers' Interpretation of Best Interests	Intensivists' Interpretation of Best Interests
2. Whether the incapable person's condition or well-being is likely to improve, remain the same or deteriorate without the treatment. 4. Whether a less restrictive or less intrusive treatment would be as beneficial as the treatment that is proposed.	1. Whether the treatment is likely to: i. improve the incapable person's condition or well being ii. prevent the incapable person's condition or well being from deteriorating iii. reduce the extent to which, or rate at which, the incapable person's condition or well being is likely to deteriorate	The person who gives or refuses consent on his or her behalf shall take into consideration a.) the values and beliefs the incapable person held when capable and believes he or she would still act on if capable b.) any wishes expressed by the incapable person with respect to treatment that are not required to followed	3. Whether the benefit the incapable person is expected to obtain from the treatment outweighs the risk of harm to him or her.

§ After Sibbald and Chidwick[3]. Numbering corresponds to the text in the HCCA.

## Methods

The institutional research ethics board approved our use of health records for this study.

The Critical Care Unit at Sunnybrook Health Sciences Centre is a 20 bed "closed unit" in which medical, surgical, and trauma patients (including surgical oncology, trauma, neurosurgery, elective surgical and internal medicine) are under the direct care of a critical care medicine team comprised of a staff intensivist, subspecialty trainees in critical care medicine, and post graduate medical and surgical trainees. Between January and June 2006, 591 patients were admitted to the Critical Care Unit. Of these, 120 (20%) died while in the critical care unit. We reviewed documentation by physicians in the charts of these 120 patients. Fourteen charts were excluded because they did not have a communication prior to their death, and 1 patient did not have a complete chart available for review. Our analysis is therefore based on the charts for 105 patients. Patient characteristics, admission diagnosis and mode of death were obtained from the charts and the Critical Care Research Network (CCRNET) database. We transcribed clinical notes handwritten by physicians prior to the patient's death. There were no electronic clinical notes in use at the time of our study. Exclusion criteria for detailed review of notes included: no documented communication before death, clinical notes limited to post mortem discharge summary, and communications with someone other than a person authorized to give or refuse consent. All patient and physician identifiers were removed from the transcribed records prior to our review.

A team of 2 intensivists and a research student developed a data collection form to discern characteristics of documented communications. Candidate domains were based on a literature search and recommendations arising from the Society of Critical Care Medicine's symposium "Improving End of Life Care in the ICU: Interventions that work" (Miami, February 17-19 2006). We evaluated and refined this data collection form (Table 2) through face and content validity checks by seeking opinions of three intensivists and a clinical ethicist not involved in the creation of the data-collection form. Ease of use and feasibility of the data collection form was evaluated through a pilot phase analysis of 10 charts. Two investigators (MR, AC) independently reviewed and scored the transcripts using the data collection form. Agreement between them was measured using percent agreement and Cohen's kappa. For discordant results, differences were subsequently resolved by consensus after kappa was calculated. We analyzed results on a per-patient basis to represent documented communication as a process up to the time of death with the exception of communication characteristics section for which the results are reported per communication. We report frequencies and percentages for discrete data, median (m) and interquartile range (IQR) for continuous data.

**Table 2:** Quality of Documented Communication — Data Collection Form with Thematic Classification following Section 21(2) of Canada's Health Care Consent Act 1996

Medical Status	Treatment Plan at Issue	Substitute Decision Makers' Interpretation of Best Interests	Intensivists' Interpretation of Best Interests
◦ Is there an explicit survival estimate? ◦ Is the diagnosis or syndrome mentioned?	◦ Are life support strategies being administered mentioned? ◦ Are options regarding future life support documented? If yes, which of: a. continuing full support b. not to escalate existing life support c. withdrawal of life support	◦ What is the patient's perspective about ICU treatment if there is a prediction of loss of function? ◦ What is the patient's perspective about ICU treatment in the context of the anticipated quality of life post hospital stay? ◦ What is the patient's perspective about ICU treatment if it will result in anticipated chronic care?	◦ Did the physician make a prediction of the patient's functional status following ICU? ◦ Did the physician make a prediction of anticipated quality of life post hospital stay? ◦ Did the physician make a prediction of the need for chronic care following discharge from the ICU?

## Results

In total, 260 clinical notes from 105 patients were available for review. Our study cohort was elderly and had a high predicted probability of death based on Acute Physiology and Chronic Health Evaluation II scores. Admission diagnoses were most commonly head trauma, cardiac arrest and subarachnoid hemorrhage (Table 3). Length of stay in the intensive care unit prior to death was short (m 2, IQR 1-5 days), and withdrawal of life support preceded death in more than half of patients (n 57, 54%). Brain death criteria were present for 18 patients (17%). Advance directives were documented for 40% of patients, but pertained to specific aspects of life support in only half of these (48%). A substitute decision maker (SDM) was explicitly recorded for only 10% of patients and we could not identify their relationship to the patient from the documentation in 42%. We found documentation of differences of opinion regarding life support between family members and the intensivist in only 10% of charts. 3.8% (4) of patients had at least one note that documented the patient's capacity for decision-making.

**Table 3:** Patient Characteristics and Admission Diagnoses

Characteristic	Mean (IQR)	
Age	72 years (58-81)	
APACHE II scores	28 (23-36)	
ICU length of stay	2 days (1-5)	
		
Diagnosis	N	**%**
Cardiac Arrest	13	12.38
Cardiogenic Shock	2	1.90
Aortic Aneurysm	1	0.95
Rhythm Disturbance	1	0.95
Other GI	2	1.90
Subarachnoid Hemorrhage	11	10.48
Intracerebral Hemorrhage	8	7.62
Laminectomy/spinal cord	1	0.95
Neuromuscular Disease	1	0.95
Seizure	1	0.95
Sepsis of Urinary Tract Origin	3	2.86
Renal Diseases	2	1.90
Respiratory Arrest	8	7.62
Other Respiratory Diseases	8	7.62
Pneumonia	4	3.81
Chronic Obstructive Pulmonary Disease	2	1.90
Pulmonary Edema (non-cardiogenic)	1	0.95
Pulmonary Embolism	1	0.95
Head Trauma (with/without multiple trauma)	24	22.86
Multiple Trauma(excluding head trauma)	3	2.86
Sepsis(other than urinary tract)	8	7.62
	105	100

Documentation by intensivists before death was timely (first note m 17, IQR 4-48 h after admission), but brief (m 51, IQR 27-78 words) (Table 4). We present the incidence of documentation for selected domains relevant to the determination of best interest (after resolving disagreements by consensus), matching our quality domains with corresponding sections of the Health Care Consent Act (Table 5).

**Table 4:** Representative Clinical Notes

Quartile (Word Count)	Representative Note
Lower (27)	"Spoke with son, provided consensus decision. No CPR, do not give cardiac shock, do not increase level of care. It was very clear supportive care will be provided."
	
Median (51)	"I met with entire family and explained patient's poor prognosis. I explained that Neurosurgery does not have anything to offer at this time. I introduced the idea of organ/tissue donation and family will discuss this. I also suggested DNR but family was not ready for this at this time."
	
Upper (78)	"Further discussion with daughter (next of kin). RN present. Updated on clinical course over previous 24 hours and re iterated that Ms. condition remains critical. Daughter feels that Ms. condition has progressively deteriorated over the previous few months and emphasized that her mother would not want further escalation of treatment/intubation +ventilation. Daughter also stated that given the multiple current issues on a background of Ms. 's pre-admission co morbidities she feels further treatment and intervention would be futile and wishes for us to withdraw care. Therefore, plan for withdrawal of care, d/c levophed infusion, comfort measures only."

**Table 5:** Documentation of Best Interest (Incidence %. N = 105 Patients)

**Medical Status**	estimate of survival likelihood (50)	diagnosis or syndrome (84)		
**Treatment Plan at Issue**	life support strategies administered (45)	future life support options (88)		
**Substitute Decision Makers' Interpretation of Best Interests**	patient's perspective about ICU treatment if there is a prediction of loss of function (18)	patient's perspectives about treatments in the context of anticipated quality of life post hospital stay (7)	patient's perspectives about treatment if chronic care is anticipated (2)	
**Intensivists' Interpretation of Best Interests**	physician's prediction of functional status following ICU (20)	physician's prediction of quality of life post hospital stay (0)	physician's prediction of the need for chronic care following discharge from the ICU (3)	

Reviewers' (AC, MR) evaluations of intensivists' documentation of domains related to diagnosis, prognosis and treatment had only moderate agreement (percent agreement 70.0% - 96.2%; kappa 0.35-0.84 Table 6). For physician and patient perspectives on post ICU functional status, chronic care and quality of life, raw agreement ranged between 73.5% -94.2%; kappa for these domains was weak (-0.011 to 0.27 Table 7), reflecting a high proportion of null results from both observers.

**Table 6:** Kappa for Intensivist Documentation of Diagnosis, Prognosis and Treatment

Variable	Cohen's Kappa (simple)	Agreement	Cohen's Kappa for clearly legible communications	Agreement for clearly legible communications
Survival Estimate	0.57	73.5%	0.84 (n = 158)	93.7%
Diagnosis	0.45	70.0%	0.57 (n = 227)	79.7%
Life support Strategies	0.35	74.6%	0.44 (n = 238)	81.5%
Future Life Support	0.48	73.1%	0.61 (n = 233)	81.5%
Family Questions	0.84	96.2%	0.87 (n = 258)	96.9%

**Table 7:** Kappa for Patient Perspectives On Post ICU Functional Status, Chronic Care And Quality Of Life

Variable	Cohen's Kappa (simple)	Agreement	Cohen's Kappa for clearly legible communications	Agreement for clearly legible communications
Intensivist Functional Status	0.13	73.5%	0.19 (n = 230)	83%
Patient Loss Function	0.27	83.5%	0.45 (n = 232)	93.5%
Intensivist Chronic Care	0.13	93.8%	0.21 (n = 251)	97.2%
Patient Chronic Dependence	0.097	84.6%	0.12 (n = 233)	93.4%
Intensivist Quality of life	-0.011	94.2%	0 (there are no communications for which both reviewers chose "Yes")	98%
Patient Quality of Life	0.16	86.2%	0.34 (n = 235)	94.9%

## Discussion

Our single-centre audit shows that intensivists' written documentation of communication with substitute decision makers frequently outlined the plan of treatment, but did not provide a justification for the plan in relation to the patient's previous wishes, or its likelihood of benefit.

We recorded low rates for physicians' predictions of functional status following ICU stay (20%), anticipated quality of life post hospital stay (0%) and prediction of need for chronic care following discharge from ICU (3%). We also found that documentation of the patient's own perspectives on these domains was low, ranging from 2-18%. Our findings are similar to those observed in a documentation review of 356 patients who died in 2 intensive care units during 2005, for whom preferences regarding life support were documented in less than one-quarter of cases [7]. However, our observations are at variance with previous studies using grounded theory methodology in a different intensive care unit. In transcribed recordings of end of life discussions between intensivists and families from a cohort of 51 trauma patients, White et al found significantly more statements about prognosis for functional outcomes, per conference, than statements about prognosis for survival [8]. In our cohort, the only items noted for more than half of our dying patients were diagnosis (84%) and options for future life support (88%). Our cohort was severely ill (APACHE II m 28), and their length of stay before death was short (m 2 days); therefore it may be have been reasonable simply to document that death was likely and that options for treatment were limited. However, withdrawal of life support occurred frequently (54%) with little accompanying documentation of whether or not physicians had communicated how continued treatment might be likely to improve the patient's condition or well-being. This pattern of practice may represent the adoption of "strong paternalism" as this group of intensivists' preferred model for the physician patient relationship. Indeed, one of the strategies currently adopted by Ontario intensivists to avoid or limit medical care that is perceived to be futile is to make the decision for the family, or refuse to accede to demands for treatment [2].

In our study, the capacity of patients to participate in treatment decisions was explicitly described in only 3.8% of the documented communications. We hypothesize that the incapability of these patients to participate in treatment decisions may have seemed obvious to clinicians due to their severe illness acuity. However, Section 20(1) of the Health Care Consent Act describes how SDMs should be identified for such incapable patients. Alarmingly, the intensivists in our study specified the relationship of substitute decision makers in a minority of patients (42%), leaving ambiguity about whether or not treatment decisions were being made by someone with legitimate authority to make them.

If conflict between clinicians and substitute decision makers over the appropriateness of life sustaining therapy cannot be resolved by improved communication, support for the burdened family, or other recommended measures, an appeal by the treating physician to a review board (for example, the Consent and Capacity Board in Ontario) may be appropriate[9]. In Ontario, the physician bringing forth such an application must demonstrate non-compliance of the SDMs with the legislative principles of substitute decision- making. In *Scardoni v. Hawryluck*, the success of the family's appeal of a CCB decision to withhold life support was based, in part, on the court's finding that the mother's pre- illness philosophy of life statement "where there's life there is hope" reflected her personal values and beliefs as specified in section 21(2) (a) of the Health Care Consent Act. The court also found that the statement formed the basis for the mother's expressed wish applicable to the circumstances as specified in section 21(2) (b) of the Act[10]. This ruling highlights the importance of carefully exploring the patient's pre-illness values, beliefs and expressed wishes in deciding how the incapable patient's best interests would be served within their particular context of illness and treatments. The court also highlighted the importance of weighing whether the incapable person's condition or well-being would improve or deteriorate as a consequence of treatment as specified in section 21(2) (c) of the Health Care Consent Act. This precedent implies that intensivists should not only explain the patient's existing condition and prognosis, but also estimate the patient's future health status (including anticipated pain and discomfort). We found almost no documentation of patients' treatment preferences if loss of function were anticipated, if quality of life might be diminished, or if complex continuing care might be required. Therefore, much of the documentation we reviewed would be insufficient grounds for an application to the Ontario Consent and Capacity Board.

While suggesting areas where documentation of communication might be improved, our results should be viewed as hypothesis generating. It is possible that a comprehensive exploration of the incapable person's best interests occured prior to death, but that recall bias or the demands of urgent care in the intensive care unit did not afford physicians sufficient time to document all that had been discussed in their family meeting. For 17 of the 20 charts excluded from our review, there was no documentation of any communication; this may reflect a system failure to capture such data in the context of physicians' present work environment. Low kappa values but high percent agreement between reviewers for domains of quality were perhaps owing to the high proportion of null results [11,12]. However, the low chance-corrected agreement more likely reflects the poor quality of documentation of communication leading to uncertainty among reviewers, since the domains of quality required subjective judgment to assign during analysis. We did not conduct training of reviewers on the relevant considerations for assigning quality domains to the data, and this has been shown to improve agreement between independent observers [13].

Our findings are from a single ICU and may not reflect the quality of documentation of end of life communications in other Canadian hospitals. However, we believe that the workload and charting challenges faced by intensivists in our hospital are not unique. We did not explore the documentation of treatment plan and determination of best interests for patients who survived to discharge from the intensive care unit, and this may represent a selection bias. Because life support was continued for these patients with longer lengths of stay, documentation of its rationale may have been, of necessity, more complete.

## Conclusion

Interventions such as proactive palliative care[14], clinical ethics consultations[15], or structured communication[16] can lead to reductions in length of ICU stay and decreases in "prolongation of dying". In our critical care unit the core elements of such interventions, for example discussions about the goals of care, were poorly documented. In situations where conflict between physicians and substitute decision makers leads to a submission for Consent and Capacity Board review, appropriate documentation of the elements required for consent to life sustaining therapy will be essential. Structured communication which follows Section 21(2) of the Health Care Consent Act may guide for clinicians to more robust documentation of the construct of best interest for the incapable, critically ill patient. Our data collection form (Table 2) may serve as a practical guide to obtain this information in the clinical setting.

We advocate for communication training for intensivists in the best interests of critically ill patients, their families and our health care system [17,18].

## Competing interests

The authors declare that they have no competing interests.

## Authors' contributions

AC, MR and DS made substantial contributions to the study concept and design, analysis and interpretation of the data, the drafting and revisions of the manuscript. MR acquired the data. RP performed all statistical analysis and proof read the final revisions. All authors have read and approved of the final manuscript.

## Pre-publication history

The pre-publication history for this paper can be accessed here:

http://www.biomedcentral.com/1472-6939/11/1/prepub
